# *FOXP3* TSDR Measurement Could Assist Variant Classification and Diagnosis of IPEX Syndrome

**DOI:** 10.1007/s10875-022-01428-w

**Published:** 2023-01-05

**Authors:** Rebecca C. Wyatt, Sven Olek, Elisa De Franco, Bjoern Samans, Kashyap Patel, Jayne Houghton, Steffi Walter, Janika Schulze, Rosa Bacchetta, Andrew T. Hattersley, Sarah E. Flanagan, Matthew B. Johnson

**Affiliations:** 1 Clinical and Biomedical Science, Faculty of Health and Life Sciences, University of Exeter, Exeter, UK; 2 Ivana Türbachova Laboratory of Epigenetics, Precision for Medicine GmbH, Berlin, Germany; 3 Exeter Genomics Laboratory, Royal Devon and Exeter NHS Foundation Trust, Exeter, UK; 4 Research & Development, Epimune Diagnostics, Berlin, Germany; 5 Department of Pediatrics, Division of Hematology, Oncology, Stem Cell Transplantation and Regenerative Medicine, Stanford University School of Medicine, Stanford, CA USA; 6 Center for Definitive and Curative Medicine (CDCM), Stanford University, Stanford, USA

**Keywords:** IPEX syndrome, FOXP3, Epigenetics, Regulatory T cells

## Abstract

Pathogenic *FOXP3* variants cause immune dysregulation polyendocrinopathy enteropathy X-linked (IPEX) syndrome, a progressive autoimmune disease resulting from disruption of the regulatory T cell (Treg) compartment. Assigning pathogenicity to novel variants in *FOXP3* is challenging due to the heterogeneous phenotype and variable immunological abnormalities. The number of cells with demethylation at the Treg cell-specific demethylated region (TSDR) is an independent biomarker of IPEX. We aimed to investigate if diagnosing IPEX at presentation with isolated diabetes could allow for effective monitoring of disease progression and assess whether TSDR analysis can aid *FOXP3* variant classification and predict disease course. We describe a large genetically diagnosed IPEX cohort (*n* = 65) and 13 individuals with other monogenic autoimmunity subtypes in whom we quantified the proportion of cells with FOXP3 TSDR demethylation, normalized to the number with CD4 demethylation (%TSDR/CD4) and compare them to 29 unaffected controls. IPEX patients presenting with isolated diabetes (50/65, 77%) often later developed enteropathy (20/50, 40%) with a median interval of 23.5 weeks. %TSDR/CD4 was a good discriminator of IPEX vs. unaffected controls (ROC-AUC 0.81, median 13.6% vs. 8.5%, *p* < 0.0001) with higher levels of demethylation associated with more severe disease. Patients with other monogenic autoimmunity had a similar %TSDR/CD4 to controls (median 8.7%, *p* = 1.0). Identifying increased %TSDR/CD4 in patients with novel *FOXP3* mutations presenting with isolated diabetes facilitates diagnosis and could offer an opportunity to monitor patients and begin immune modulatory treatment before onset of severe enteropathy.

**Supplementary Information:**

The online version contains supplementary material available at 10.1007/s10875-022-01428-w.

## Introduction

Immune dysregulation, polyendocrinopathy, enteropathy, X-linked (IPEX) syndrome are caused by pathogenic variants in *FOXP3*, a transcription factor critical for the development of functional regulatory T-cells (T_regs_) [1]. IPEX patients usually present with rapidly progressing multiple autoimmunity, including autoimmune diabetes (typically neonatal diabetes; NDM; onset < 6 months), life-threatening enteropathy, and atopic dermatitis. It is this classic triad of symptoms that is often the catalyst for genetic testing. However, the clinical, immunological, and molecular phenotype is heterogeneous, and increasingly, patients are reported with atypical features or a milder clinical course [2–4]. For progressive or syndromic genetic disorders such as IPEX syndrome, an early and accurate genetic diagnosis can provide insight into prognosis and direct monitoring and treatment plans, often improving outcomes [5].

Gene panel testing has revolutionized genetic testing and highlighted the phenotypic variability associated with monogenic diseases such as IPEX syndrome, with pathogenic variants being reported in individuals without all the classic features of the genetic disorder [6–8]. In such cases, assigning pathogenicity to novel variants can be difficult and may rely on waiting for additional features to develop. Isolated NDM may be the first and only feature at testing for IPEX, which overlaps with > 30 other monogenic disorders including other “Tregopathies” (sometimes termed IPEX-like disorders) [3, 9, 10]. Variant interpretation is further complicated by a high proportion of maternally inherited variants making co-segregation analysis difficult and an alternatively spliced isoform of *FOXP3* which makes variants in exon 2 challenging to interpret.

Functional immune analyses can assist with understanding the impact of a *FOXP3* variant. However, these techniques require fresh blood samples to be rapidly processed by specialist laboratories, which can be prohibitive due to cost, expertise needed, and remoteness of patients to the laboratory [10, 11]. Quantitative assessment of immune cells by methylation-specific qPCR is an alternative option for the analysis of human leukocyte populations and can be used to quantify different cell types in whole-blood derived DNA [12]. This cost-effective method does not rely on fresh samples and can be easily performed in a genomics laboratory using existing samples taken for genetic testing. The T_reg_ cell-specific demethylated region (Foxp3 TSDR) is an imprinted region within the *FOXP3* locus that controls and stabilizes expression of *FOXP3.* De-methylation of the TSDR allows for stable *FOXP3* expression and subsequent maintenance of T_reg_ identity [13]. T_reg_ count by flow cytometry is very tightly correlated with the number of cells with demethylation of the TSDR in healthy individuals but appears to be unphysiologically high in individuals with IPEX [14].

We report a large cohort of patients with IPEX syndrome (*n* = 65) resulting from a hemizygous *FOXP3* pathogenic variant identified by custom gene panel testing for monogenic NDM. We assessed whether genetically diagnosing IPEX at presentation with isolated NDM could offer a window in which to monitor and intervene before patients become critically ill. We then sought to assess if the number of cells characterized by a demethylated *FOXP3* TSDR could have diagnostic and prognostic value in atypical IPEX.

As described in the method section, we determined the number of blood cells with demethylated FOXP3 TSDR or a region in the CD4 locus specifically demethylated in T helper cells (demethylated CD4). These counts were initially analyzed relative to the number of all nucleated blood cells determined by the number of demethylated copies of a region of the GAPDH gene previously been shown to be demethylated in all cells (demethylated GAPDH). Based on these counts, we quantified the percentage of Tregs (% demethylated FOXP3 TSDR) and CD4 + T helper cells (% demethylated CD4) in leukocytes as well as the percentage of Tregs within the T helper cells (% demethylated FOXP3 TSDR/demethylated CD4 + , henceforth referred to as %TSDR/CD4).

## Methods

### Cohorts

All individuals were referred for research-funded genetic testing to the Exeter Genomics Laboratory between 2000 and 2021. Non-IPEX control samples were either unaffected siblings sent for predictive testing who subsequently were found not to have inherited the genetic variant (*n* = 10) or individuals with neonatal diabetes (NDM) due to a pathogenic KATP variant (*n* = 19) (i.e., non-autoimmune). IPEX patients and individuals with other IPEX-like monogenic autoimmunity were referred for genetic testing with either isolated NDM or with a clinical suspicion of monogenic autoimmunity. Individuals with a *FOXP3* variant of uncertain significance were referred for genetic testing for isolated neonatal diabetes.

### Genetic Testing

Sequencing was undertaken either by rapid Sanger sequencing of *FOXP3* or targeted next-generation sequencing of all genes known to cause neonatal diabetes or monogenic autoimmune diabetes. Full details of this testing can be found at https://www.diabetesgenes.org/tests-for-diabetes-subtypes/targeted-next-generation-sequencing-analysis-of-45-monogenic-diabetes-genes/. Novel variants were classified according to the ACGS best practice guidelines for variant classification [15].

### Epigenetic qPCR

Pseudonymized whole-blood-derived extracted DNA was tested at Epimune GmbH using the previously published qPCR protocol with amplicons specific for the TSDR and CD4 loci [14]. Data were unblinded and analyzed at the University of Exeter Medical School. Epigenetic quantitative real-time PCR (qPCR) assays targeting demethylated CpGs (as converted to “UpC” and after amplification “TpGs”) were developed for the Foxp3 and CD4 regions, as bisulfite-sequencing revealed that these regions were specifically demethylated in the respective target cell populations, i.e., Tregs and T helper cells, respectively [14, 16]. Using these assays, DNA copy numbers and relative cell counts in a sample were determined. Copy numbers were calculated based on an internal standard curve resulting from the parallel measurement of a serially diluted in silico bisulfite converted plasmid that contains the assay target sequences for TSDR, CD4, and GAPDH. Due to assay-specific and bisulfite conversion-specific differences in performance efficiencies, the uncalibrated relative cell count was normalized using an assay-specific calibration factor. This calibrator was based on a plasmid containing the genomic assay target sequences. It is treated, amplified, and analyzed in parallel with the samples, and the resulting TpG and GAPDH copy number is used to calculate the calibration factor (calibration factor = TpG_calibrator_ [copies]/GAPDH_calibrator_ [copies]). A detailed description of this process was published by Baron colleagues (STM, 2018). The target percent cell count (expressed as [% demethylated Foxp3 TSDR] or [% (% demethylated CD4 + T cells)]) in a sample is given by the quotient of the cell type-specific (i.e., Foxp3 TSDR or CD4) and GAPDH-specific demethylation multiplied by 100. The percentage share of Treg cells within CD4 + T cells is determined by the adjusted values of the demethylated Foxp3 TSDR per demethylated CD4.

### Statistical Analyses

We used the ANOVA test for multiple comparison of continuous variables and Fisher’s exact for categorical variables. Associations between TSDR/CD4 and age at sampling or symptom duration were measured using simple linear regression. The performance of TSDR/CD4 in discriminating health from disease was analyzed using the area under the receiver-operator characteristics curve (ROC-AUC). For all analyses, a two-tailed *p* value of ≤ 0.05 was considered significant.

## Results

### Identifying Pathogenic FOXP3 Variants in Patients Presenting with NDM Provides a Window of Opportunity to Monitor and Treat

We report 65 males with 39 different pathogenic or likely pathogenic *FOXP3* variants, 16 of which are novel and were classified according to ACGS best practice guidelines (ESM Table 1) [15]. Where a maternal sample was available, 50/55 (91%) patients had inherited the variant from their unaffected mother. In the remaining 5 cases, the variant had arisen de novo.

The presenting feature was diabetes in 50/65 (77%) patients. Of those, 47/50 (95%) presented with NDM and 3/50 (5%) were diagnosed aged between 6 months and 1 year. The remaining 15 presented with enteropathy and/or other features (ESM Table 1, ESM Fig. 1). Of the 50 who presented with diabetes, 20 (40%) are known to have developed enteropathy (i.e., have classical IPEX) during follow-up (median follow up 48 weeks, [IQR 17.2–329 weeks]). The median time from diabetes onset to enteropathy was 23.5 weeks [IQR 7.8–126.8 weeks]. No individuals were on immunosuppressive therapy at the time of sampling; individuals with diabetes were treated with insulin.

### TSDR/CD4 Measurement Can Distinguish Between NDM Patients with an IPEX-Causing FOXP3 Variant, Healthy Controls, and Individuals with Other Tregopathies

**Fig. 1:**
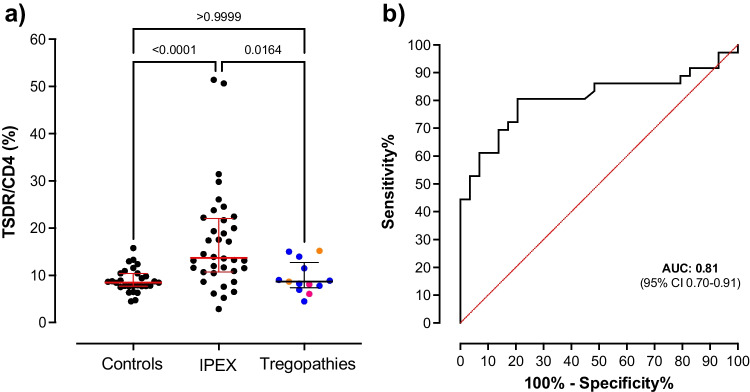
**a** %TSDR/CD4 in controls (*n* = 29, median 8.5%, IQR 7.7–10.4%) compared with patients with IPEX (*n* = 41, median 13.6%, IQR 10.5–22.3, *p* < 0.0001) and patients with overlapping IPEX-like phenotypes due to other monogenic Tregopathies (*n* = 13, median 8.7%, IQR 7.4–12.7, *p* = 1.0; blue dots represent patients with homozygous pathogenic *LRBA* variants, red dots individuals with homozygous pathogenic *IL2RA* variants, and yellow dots individuals with gain-of-function *STAT3* variants [see ESM Table 3 for detailed information]). Horizontal lines represent the median and interquartile range. **b** Receiver-operator characteristic curve for TSDR/CD4 based on data from 41 IPEX patients and 29 controls. The area under the curve (AUC) was 0.81

In 41 genetically confirmed IPEX patients where there was sufficient DNA, we next measured the % demethylated FOXP3 TSDR to assess if it could aid in rapid diagnosis. The median duration of disease at sampling was 11.2 weeks (IQR 3.6–83.1). We found that these individuals had slightly reduced demethylation at the % demethylated CD4 and increased % demethylated Foxp3 TSDR (both as a percentage of demethylated GAPDH from whole blood) compared to healthy controls (ESM Fig. 2). Levels of % demethylated FOXP3 TSDR as a proportion of % demethylated CD4 (%TSDR/CD4) were therefore higher (median 13.6% [IQR 10.5–22.3]) compared with male controls (*n* = 29; median TSDR/CD4 8.5%, [IQR 7.7–10.4], *p* < 0.0001) or males with other IPEX-like Tregopathies (*n* = 13, median TSDR/CD4 8.7% [IQR 7.4–12.7], *p* = 0.01) (Table 1, Fig. 1; ESM Table 1, Table 3).

**Table 1:** Cohort characteristics. *W*, weeks; *IQR*, interquartile range; *N/A*, not applicable

	IPEX (*n* = 41)	Controls (*n* = 29)	Tregopathies (*n* = 13)
Median sampling age, w (IQR)	20.7 (7.5–115.9)	26.6 (120–127.3)	42.3 (24.1–70.3)
Median age at onset, w (IQR)	3 (1–7.5)	N/A	24 (2–37)
Median duration, w (IQR)	11.2 (3.6–83.1)	N/A	29.9 (2.4–76.0)
Median follow-up, w (IQR)	8 (0–23)	N/A	N/A
Median CD4, % (IQR)	15.4 (11.9–20.3)	20 (14–32)	20 (16–24)
Median T_reg_, % (IQR)	2.4 (1.8–3.3)	2 (1–3)	1.7 (1–2.2)
Median TSDR/CD4, % (IQR)	13.6 (10.5–22.3)	8.5 (7.7–10.4)	8.7 (7.4–12.7)

**Fig. 2:**
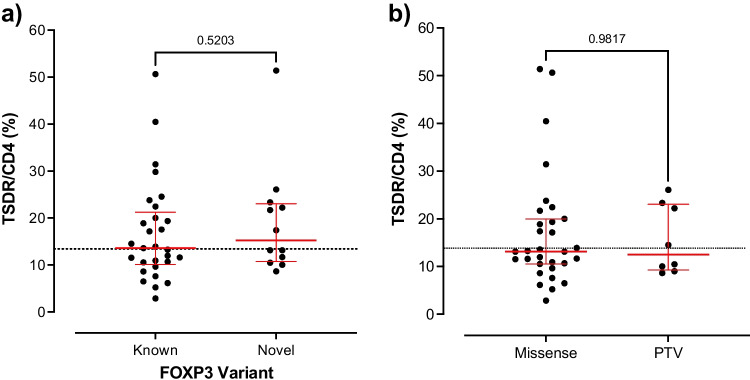
**a** %TSDR/CD4 values in patients with either previously reported (“known”) or novel variants. The dotted line is the cut off of 13.4%. **b** %TSDR/CD4 values in individuals with either a missense or protein truncating variant (“PTV”)

We conducted receiver-operator characteristic analysis on our control and IPEX cohort to assess the discriminatory ability of the %TSDR/CD4 and identify the optimal threshold for discrimination. The overall area under the curve was 0.81 [95% CI 0.71–0.91] and the optimal cut-off based on 95% specificity was 13.4%, which had 51% sensitivity to identify IPEX patients (Fig. 1b). This supports that %TSDR/CD4 is a useful tool to aid diagnosis in individuals with a *FOXP3* variant of uncertain significance. For 11 out of 16 patients with novel Foxp3 mutation variants, the percentage of TSDR/CD4 was available. Six of these patients (55%) also showed an increased %TSDR/CD4 (value greater than or equal to 13.4%) (Fig. 2a).

To assess whether *FOXP3* genotype and the %TSDR/CD4 were related, we compared values in patients with missense variants (*n* = 31, median 13.1% [IQR 10.6–20.0%]) and protein truncating variants (*n* = 8, median 12.5% [IQR 9.3–23.1]). These values were similar, although more variable, in individuals with missense variants regardless of whether they were in the forkhead box domain (which is where the majority of pathogenic variants cluster) or not (Fig. 2b).

### Case Study: Use of %TSDR/CD4 in Determining Pathogenicity of a Novel FOXP3 Variant

**Fig. 3:**
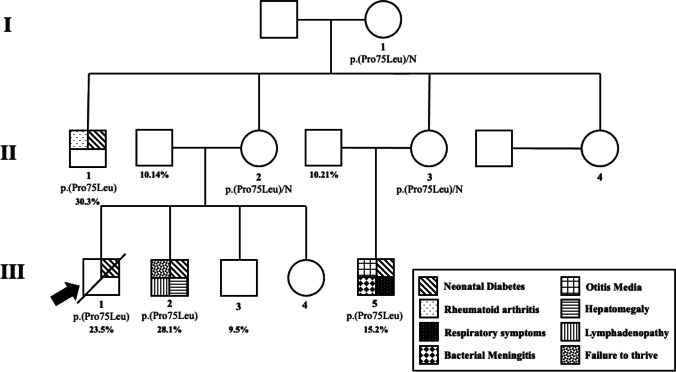
Partial pedigree of a family with novel *FOXP3* variant in the alternatively spliced exon two (p.Pro75Leu). Arrow denotes proband. Frequencies refer to %TSDR/CD4

We found that determining the %TSDR/CD4 was useful in interpreting a case without classic IPEX features who had a novel missense variant in the alternatively spliced exon two of *FOXP3*, p.(Pro75Leu) (individual not included in main analysis; Fig. 3, ESM Table 4).

During our study, a male patient from Palestine was referred for genetic testing after developing isolated diabetes aged 6 months (insulin treated) and presenting with severe diabetic ketoacidosis aged 5 years resulting from lack of access to insulin which was subsequently fatal (patient III.1). Sequencing of *FOXP3* revealed this patient had a novel missense variant in the alternatively spliced exon two, p.(Pro75Leu), which was originally classified as a VUS by ACMG guidelines [15]. We subsequently measured %TSDR/CD4 and found them to be more than double the mean value in controls (23.5% vs. 10.1%), providing evidence to suggest the variant was causal. On further discussion with the clinician, we established that there was a family history of early onset diabetes or immunodysregulatory features affecting his brother, male cousin, and an uncle. No family member had enteropathy, the most common and severe feature of IPEX syndrome. We were then able to confirm co-segregation of the variant, establish that %TSDR/CD4 values were raised in the affected (30.3%, 23.5%, 28.1%, and 15.2%) but not unaffected (10.21, 10.14, and 9.5%) male relatives, and establish pathogenicity using ACMG guidelines. The two younger affected relatives of the proband have now be referred for hematopoietic stem cell transplantation (HSCT).

### %TSDR/CD4 May Offer Insight into IPEX Severity in Patients Who Presented with Diabetes

**Fig. 4:**
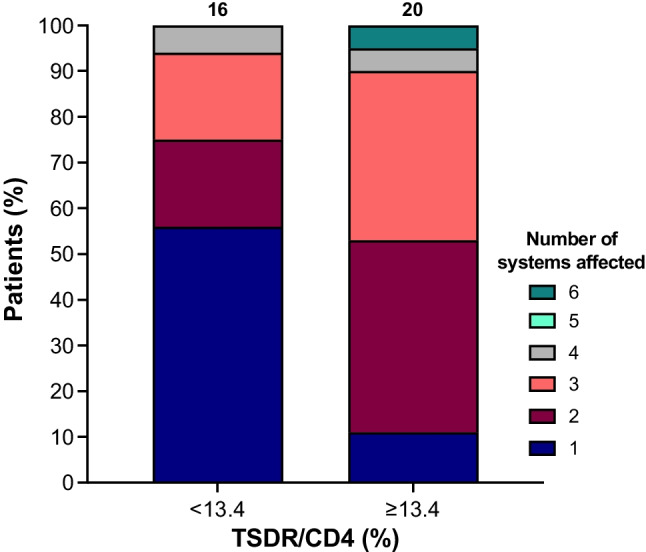
Proportion of IPEX patients with 1, 2, 3, 4, 5, or 6 systems affected in those with a TSDR/CD4 value of < 13.4% (*n* = 16) and $\ge$ 13.4% (*n* = 19). Of those with a %TSDR/CD4 $\ge$ 13.4%, 17 (89%) had $\ge$ 2 systems affected by the end of follow-up compared with 7 (44%) in those with a %TSDR/CD4 < 13.4%. *p* = 0.009

To assess the prognostic potential of the %TSDR/CD4, we looked at clinical features developed during follow-up by patients who had initially presented with diabetes. We categorized additional features according to the system/organ affected (see ESM Table 5 for categories). Using the cut off of 13.4%, we assessed whether the number of affected systems differed between patients who presented with diabetes and had %TSDR/CD4 values above or below the threshold (Fig. 4). Those with a higher %TSDR/CD4 were more likely to have $\ge$ 2 systems affected ($\ge$ 13.4% 17/19 [89%] vs. < 13.4% 7/16 [44%], *p* = 0.009). The %TSDR/CD4 may therefore offer insight into IPEX severity, though we were unable to determine if this reflects underlying pathophysiology or is a consequence of severity. The median age at latest follow-up did not differ between the two groups (ESM Fig. 3) but was relatively short (< 13.4%: *n* = 15, median 26.1 weeks, IQR 13–886 weeks vs $\ge$ 13.4%: *n* = 18, median 43.5 weeks, IQR 19.8–156.3 weeks, *p* = 0.6), and some patients may have subsequently developed additional features.

We do not suggest that TSDR/CD4 should be used to guide clinical practice as this data are not longitudinal and a high proportion of patients with TSDR/CD4 below 13.4% have ≥ 2 systems affected. While this tool appears to add understanding to clinicians for some patients, the lack of full longitudinal studies means caution is required in interpreting the data. In a disease as rare as IPEX, potentially valuable data should be monitored but, due to the lack of population studies, interpreted cautiously.

## Discussion

We have shown, in the largest single-center series of IPEX patients, that identifying and accurately diagnosing IPEX early at initial presentation with infancy-onset diabetes could offer an opportunity to monitor patients before the onset of life-threatening features. Measurement of demethylation at the T-reg specific demethylated region (TSDR), a specific marker of thymic derived regulatory T cells [17], represented as a proportion of all T helper cells (as measured by demethylation at a CD4 T helper cell specific demethylated region) could be useful for interpreting novel variants of uncertain significance in *FOXP3*, facilitating early diagnosis where clinical features are not sufficiently informative (i.e., patients have isolated diabetes). The %TSDR/CD4 was raised in individuals with IPEX compared to healthy controls, individuals with non-autoimmune monogenic neonatal diabetes, and non-IPEX monogenic autoimmunity with overlapping phenotype. In addition, we found evidence that a higher %TSDR/CD4 value correlates with the severity of phenotype in our cohort, suggesting this may be a marker of disease severity.

We found the presenting feature was diabetes in 77%. This is higher than previous reports and likely reflects referral bias as we offer research-funded genetic testing to any individual diagnosed with diabetes before 6 months (www.diabetesgenes.org). Diagnosing IPEX rapidly after presentation with isolated NDM allows time for additional monitoring, for example, by testing for diagnostic autoantibodies (e.g., antienterocyte autoantibodies for enteropathy), which can appear before overt clinical disease [1]. It may therefore allow patients immunosuppressive treatment options (e.g., Sirolimus) that delay or reduce the development of additional, life-threatening symptoms. Currently, hematopoietic stem cell transplantation (HSCT) is the only curative therapy for IPEX, and survival after HSCT is significantly impacted by disease severity [18]. Amelioration of insulin dependency has been reported in an adolescent treated with HSCT 1 month after diabetes onset [19], suggesting the period from onset may also impact efficacy. This further underlines the importance of rapid genetic testing and variant classification. We found that individuals with a higher %TSDR/CD4 had more systems affected.

In healthy subjects, the number of DNA copies demethylated at the TSDR faithfully reflects the number of functional (i.e., suppressive) Tregs and measurement correlates closely with quantification of Tregs from flow cytometry data [14, 20]. A similar association was also found between demethylation of the CD4 locus and the number of CD4 + T helper cells, among others [14, 16]. However, upon mutation of the Treg-defining FOXP3 gene and protein, the association is disrupted. In individuals with IPEX syndrome, functional Tregs are largely absent, and one of the most typical Treg markers, intracellular antibody staining of FOXP3, may no longer work [21]. At the same time, the number of cells counted by other Treg surface markers (e.g., CD4( +), CD25( +), and CD127(low)) may no longer reflect fully functional Tregs [1, 10, 21, 22] which are highly variable in individuals with IPEX syndrome [23]. Indeed, a breakdown of correlation between surface Treg markers and *FOXP3* TSDR demethylation was recently shown in 15 IPEX patients whose FOXP3 expression was independent of TSDR demethylation [23]. We were not able to measure regulatory T cell number or function in our cohort to assess whether TSDR quantification is related to Treg function in our cohort, as we were not able to obtain fresh samples for analysis.

We have demonstrated the utility of an established and relatively inexpensive test (methylation specific qPCR) which can aid *FOXP3* variant classification and offer some insight into severity of IPEX symptoms. The utility of this measurement is demonstrated by our patient with a novel missense variant in the alternatively spliced exon two who had isolated diabetes at age 5 years but in whom the TSDR/CD4 value was raised leading to reclassification of the variant as likely pathogenic. Critically, measuring TSDR/CD4 does not require additional samples from patients and can even be run on dried blood spots [14]. It is important to note, however, that our data are cross sectional with variable follow-up periods and rely on comprehensive reporting of symptoms by referring clinicians. A recent study on 15 individuals with IPEX syndrome showed that demethylation at the TSDR increased gradually with disease progression, having normal values in new-born blood spot samples from individuals with pathogenic *FOXP3* variants that increased as IPEX syndrome developed in early life [23].

Previous studies have shown that the epigenetic measurement of lineage-specific differentially methylated loci may be useful for quantifying the immune system in individuals with primary immune disorders. This contrasts to individuals with IPEX where demethylation of the TSDR is unphysiologically high. In a recent pre-printed study currently under review, a population of cells from 4 IPEX patients were shown to have demethylated FOXP3 TSDR but not markers of Treg phenotype (CD25 and FOXP3). These cells originated both from the effector T cell compartment and from “regulatory T cells” that, due to the *FOXP3* mutation, gained effector functions such as the secretion of Th17 and Th2 cytokines [24]. This provides tantalizing evidence for the underlying mechanism for the increased %TSDR/CD4 in IPEX syndrome we have shown. A previous study showed reduced cells with TSDR demethylation (i.e., lower “Treg” as measured by methylation analysis) in individuals with non-IPEX Tregopathies that did not correlate with flow cytometric analysis of FOXP3 positive cells [25]. This is in contrast to our data where individuals with other Tregopathies had similar TSDR/CD4 ratios to healthy controls.

Identifying IPEX at initial presentation with diabetes offers a critical window of time to begin monitoring patients and intervening if necessary. All males with NDM should have rapid comprehensive genetic testing that includes *FOXP3*. Measurement of %TSDR/CD4 could be an important additional tool to aid classification of variants in IPEX syndrome facilitating early diagnosis and improved outcomes, particularly in cases with atypical clinical features. Importantly, this biomarker seems to be specific to IPEX syndrome rather than a general marker of Treg insufficiency, which is common in other monogenic autoimmune conditions. Finally, we found that %TSDR/CD4 may be linked to severity; therefore, further longitudinal studies are warranted.

## Supplementary Information

Below is the link to the electronic supplementary material.

Supplementary file1 (DOCX 335 KB)

## Data Availability

The genotype and clinical data in this study could be used to identify individuals and so cannot be made openly available. Access to data is open to any scientist or institution that complies with the required data protection regulation to protect the identity of the donors, within the framework of the existing consent. Requests for collaboration can be made by application to the Genetic Beta Cell Research Bank (https://www.diabetesgenes.org/current-research/genetic-beta-cell-research-bank/).
