# Supplementary material for: FOXP3 TSDR Measurement Could Assist Variant Classification and Diagnosis of IPEX Syndrome
Source: J Clin Immunol. 2023 Jan 5;43(3):662–9. doi: 10.1007/s10875-022-01428-w (PMC9957900; doi:10.1007/s10875-022-01428-w)
Supplement: Supplementary file 1 — Supplementary file1 (DOCX 335 KB) [file 10875_2022_1428_MOESM1_ESM.docx]

# Electronic Supplementary Material

# *FOXP3* TSDR measurement could assist variant classification and diagnosis of IPEX syndrome

Rebecca C. Wyatt^1^, Sven Olek^2^, Elisa De Franco^1^, Bjoern Samans^2^, Kashyap Patel^1^, Jayne Houghton^3^, Steffi Walter^4^, Janika Schulze^4^, Rosa Bacchetta^5, 6^, Andrew T. Hattersley^1^, Sarah E Flanagan^1^ & Matthew B. Johnson^1^

1. Institute of Biomedical and Clinical Science, University of Exeter Medical School, UK
2. Ivana Türbachova Laboratory of Epigenetics, Precision for Medicine GmbH, Berlin, Germany
3. Exeter Genomics Laboratory, Royal Devon and Exeter NHS foundation trust, UK
4. Research & Development, Epimune Diagnostics, Berlin, Germany
5. Department of Pediatrics, Division of Hematology, Oncology, Stem Cell Transplantation and Regenerative Medicine, Stanford University School of Medicine, Stanford, CA, United States
6. Center for Definitive and Curative Medicine (CDCM), Stanford University, United States

# TABLES

***ESM Table 1:*** Characteristics and variant references of patients with IPEX-causing *FOXP3* variants.

HSCT – haemotopoietic stem cell transplant. D -days. W – weeks. Mo - months Y- years.

| **ID** | **NM_014009.4 (FOXP3) variant** | **Exon/**  **Intron** | **Domain** | **Protein Effect** | **First feature**  **(age)** | **Clinical features at testing**  **(age)** | **Clinical features at end of follow-up (age)** | **Systems affected**  **(n)** | **Variant Reference** |
| --- | --- | --- | --- | --- | --- | --- | --- | --- | --- |
| **01** | c.1040G>A  p.(Arg347His) | 10 | Fork-head | Missense | Diabetes  **(1d)** | Diabetes  **(17d)** | Diabetes  **(17d)** | 1 | [1-4] |
| **02** | c.748_750del p.(Lys250del) | 8 | Leucine zipper | In-Frame  Deletion | Diabetes  **(20w)** | Diabetes, severe atopic dermatitis  **(1y)** | Diabetes, severe atopic dermatitis  **(1y)** | 2 | [5-9] |
| **03** | c.816+7G>C  p.(?) | 8 | Splice site | Splice Site | Diabetes  **(32d)** | Diabetes, kidney disease  (**Deceased age unknown**) | Diabetes, kidney disease  **Deceased (unknown)** | 2 | [10] |
| **04** | c.1040G>A  p.(Arg347His) | 10 | Fork-head | Missense | Diabetes  **(20w)** | Diabetes, coeliac disease  **(15y)** | Diabetes, coeliac disease  **(15y)** | 2 | [1-4] |
| **05** | c.1010G>A  p.(Arg337Gln) | 10 | Fork-head | Missense | Diabetes  **(2w)** | Diabetes, enteropathy, growth hormone deficiency, epilepsy  **(16y)** | Diabetes  **(HSCT aged 19y)** | 1 | [8, 11-13] |
| **06** | c.1163A>G  p.(Asn388Ser) | 12 | Fork-head | Missense | Diabetes  **(10d)** | Diabetes  **(2w)** | Diabetes, diarrhea  **(Deceased aged ~6mo)** | 2 | [14] |
| **07** | c.1150G>A  p.(Ala384Thr) | 12 | Fork-head | Missense | Diabetes  **(14d)** | Diabetes, mild enteropathy, Asthma, prurigo nodularis  **(26y)** | Diabetes, pneumonia, malnutrition, gastroparesis, hypothyroidism, hypopituitarism with growth failure, hypogonadism, premature aging, severe asthma, severe diarrhea, atopic dermatitis  **(28y)** | 4 | [15, 16] |
| **08** | c.1010G>A  p.(Arg337Gln) | 10 | Fork-head | Missense | Diabetes  **(4w)** | Diabetes  **(33d)** | Diabetes, enteropathy, atopic dermatitis  **(Deceased aged 45w)** | 3 | [8, 11-13] |
| **09** | c.1040G>A  p.(Arg347His) | 10 | Fork-head | Missense | Diabetes/  Anaemia  **(4w)** | Diabetes, severe anaemia, abnormal LFTs, respiratory insufficiency  **(2.5mo)** | Diabetes, severe anaemia, abnormal LFTs, respiratory insufficiency  **(Deceased, aged <4mo)** | 3 | [1-4] |
| **10** | c.1150G>A  p.(Ala384Thr) | 12 | Fork-head | Missense | Diabetes  **(8d)** | Diabetes  **(2mo)** | Diabetes  **(2mo)** | 1 | [15, 16] |
| **11** | c.1040G>A  p.(Arg347His) | 10 | Fork-head | Missense | Diabetes  **(4w)** | Diabetes, anaemia  **(5w)** | Diabetes, anaemia  (**5w**) | 2 | [1-4] |
| **12** | c.748_750del  p.(Lys250del) | 8 | Leucine zipper | In-Frame  Deletion | Diabetes  **(9d)** | Diabetes, hepatomegaly, history of anaemia  **(18mo)** | Diabetes, hepatomegaly, history of anaemia  **(2y)** | 3 | [5-9] |
| **13** | c.1222G>A  p.(Val408Met) | 12 | Fork-head | Missense | Diabetes  **(6w)** | Diabetes  **(20w)** | Diabetes  **(25w)** | 1 | [8, 11] |
| **14** | c.1040G>A  p.(Arg347His) | 10 | Fork-head | Missense | Diabetes  **(unknown)** | Diabetes  **(1mo, 20d)** | Diabetes  **(12w)** | 1 | [1-4] |
| **15** | c.1190G>A  p.(Arg397Gln) | 12 | Fork-head | Missense | Diabetes  **(8d)** | Diabetes  **(12d)** | Diabetes  **(13w)** | 1 | [17, 18] |
| **16** | c.1010G>A  p.(Arg337Gln) | 10 | Fork-head | Missense | Diabetes  **(7d)** | Diabetes  **(13d)** | Diabetes  **(13d)** | 1 | [8, 11-13] |
| **17** | c.323C>T  p.(Thr108Met) | 4 | N-terminal | Missense | Diabetes  **(24w)** | Diabetes  **(7mo)** | Diabetes  **(7mo)** | 1 | [19] |
| **18** | c.1150G>A  p.(Ala384Thr) | 12 | Fork-head | Missense | Hypothyroidism  **(Unknown)** | Hypothyroidism, diabetes, exfoliative dermatitis  **(4mo)** | Hypothyroidism, diabetes, exfoliative dermatitis  **(Deceased, aged 4mo)** | 2 | [15, 16] |
| **19** | c.1222G>A  p.(Val408Met) | 12 | Fork-head | Missense | Diabetes  **(3w)** | Diabetes, hypothyroidism, recurrent diarrhea  **(9y)** | Diabetes, hypothyroidism, recurrent diarrhea  **(12y)** | 2 | [8, 11] |
| **20** | c.1040G>A  p.(Arg347His) | 10 | Fork-head | Missense | Diabetes  **(1d)** | Diabetes, coeliac disease, late onset puberty, slightly elevated LFTs  **(36y)** | Diabetes, coeliac disease, late onset puberty, slightly elevated LFTs  **(36y)** | 3 | [1-4] |
| **21** | c.1010G>A  p.(Arg337Gln) | 10 | Fork-head | Missense | Diabetes  **(18mo)** | Diabetes, hydronephrosis and malrotation renis sin, hyper IgE, recurrent infections, eosinophilia, immune deficiency with severe bacterial infections, atopic dermatitis, enteropathy  **(Deceased, aged 2.5y)** | Diabetes, hydronephrosis and malrotation renis sin, hyper IgE, recurrent infections, eosinophilia, immune deficiency with severe bacterial infections, atopic dermatitis, enteropathy  **(Deceased, aged 2.5y)** | 6 | [8, 11-13] |
| **22** | c.751_753del  p.(Glu251del) | 8 | Leucine zipper | In-Frame  Deletion | Diabetes  **(13d)** | Diabetes, enteropathy, nephrotic syndrome, raised IgE.  **(4.5y)** | Diabetes, enteropathy, nephrotic syndrome, raised IgE.  **(4.5y)** | 4 | [8, 19] |
| **23** | c.1150G>A  p.(Ala384Thr) | 12 | Fork-head | Missense | Enteropathy  **(2.5mo)** | Diabetes, enteropathy, skin lesions  **(3mo)** | Diabetes, enteropathy, skin lesions  **(3mo)** | 3 | [15, 16] |
| **24** | c.1240del  p.(Arg414fs) | 12 | Fork-head | Frameshift | Diabetes  **(7w)** | Diabetes  **(2mo)** | Diabetes  **(13w)** | 1 | **Novel** |
| **25** | c.1153A>C  p.(Ile385Leu) | 12 | Fork-head | Missense | Diabetes  (**1d**) | Diabetes, evidence of malabsorption, anaemia,  **(4mo)** | Diabetes, evidence of malabsorption,  steatorrehea and diarrhea, **(Deceased, aged 10mo)** | 2 | **Novel** |
| **26** | c.991T>G  p.(Phe331Val) | 10 | Other | Missense | Diabetes  **(13w)** | Diabetes  **(8mo)** | Diabetes, enteropathy, hypothyroidism, pneumonia  **(Deceased, 10mo)** | 3 | **Novel** |
| **27** | c.543-1G>T  p.(?) | 5 | Splice site | Splice site | Diabetes  **(1w)** | Diabetes, anaemia, neonatal cholestasis, cardiac defect  **(2mo)** | Diabetes, anaemia, neonatal cholestasis, cardiac defect, feeding intolerance, suspected pancreatic agenesis, no weight gain  **(3mo)** | 3 | **Novel** |
| **28** | c.543-1G>T  p.(?) | 5 | Splice site | Splice site | Diabetes  **(6w)** | Diabetes, anaemia  **(56d)** | Diabetes, enteropathy, atopic dermatitis  **(19w)** | 3 | **Novel** |
| **29** | c.1087A>C  p.(Ile363Leu) | 11 | Fork-head | Missense | Diabetes  **(6w)** | Diabetes, abnormal LFTs, anaemia  **(27y)** | Diabetes, abnormal LFTs, anaemia  **(28y)** | 3 | **Novel** |
| **30** | c.1236G>C  p.(Glu412Asp) | 12 | Fork-head | Missense | Diabetes  **(48w)** | Diabetes, nephrotic syndrome  **(15mo)** | Diabetes, nephrotic syndrome  **(16mo)** | 2 | **Novel** |
| **31** | c.1133C>T  p.(Pro378Leu) | 11 | Fork-head | Missense | Diabetes  **(12d)** | Diabetes, anaemia, thyroid dysfunction  **(12d)** | Diabetes, anaemia, thyroid dysfunction  **(20w)** | 2 | [20] |
| **32** | c.1282dup  p.(Thr428fs) | 12 | Other | Frameshift | Diabetes  **(5w)** | Diabetes, anaemia  **(36d)** | Diabetes, anaemia  **(24w)** | 2 | **Novel** |
| **33** | c.1073G>A  p.(Arg358Gln) | 10 | Fork-head | Missense | Diabetes  **(16d)** | Diabetes  **(2w)** | Diabetes  **(6mo)** | 1 | **Novel** |
| **34** | c.766A>G  p.(Met256Val) | 8 | Other | Missense | Diabetes  **(10mo)** | Diabetes  **(9y)** | Diabetes, nephrotic syndrome, mild eczema  **(17y)** | 3 | [21] |
| **35** | c.542G>C  p.(Ser181Thr) | 5 | N-terminal | Missense | Diabetes  **(Birth)** | Diabetes  **(11d)** | Diabetes, poor weight gain, episode of bloody stool & eosinophilia, severe eczema, milk protein allergy  **(33w)** | 3 | **Novel** |
| **36** | c.1150G>A  p.(Ala384Thr) | 12 | Fork-head | Missense | Diabetes  **(3w)** | Diabetes  **(11mo)** | Unknown  **(2y – HSCT)** | 1 | [15, 16] |
| **37** | c.1190G>A  p.(Arg397Gln) | 11 | Fork-head | Missense | Enteropathy  **(4mo)** | Enteropathy  **(5mo)** | Enteropathy  **(5mo)** | 1 | [17, 18] |
| **38** | c.1150G>A  p.(Ala384Thr) | 11 | Fork-head | Missense | Enteropathy  (**Unknown**) | Enteropathy  (**Unknown**) | Enteropathy  (**Unknown**) | 1 | [15, 16] |
| **39** | c.1037T>C  p.(Ile346Thr) | 10 | Fork-head | Missense | Enteropathy  **(Unknown)** | Enteropathy, severe dermatitis, fungal infections  **(3mo)** | Enteropathy, severe dermatitis, fungal infections  **(3mo)** | 3 | [8] |
| **40** | c.-23G>A  (p.?) | 1 | Splice site | Splice site | ND  **(Unknown)** | “Work-up consistent with IPEX, reduced FOXP3 expression, anti-enterocyte antibodies  **(34w)** | Waiting for HSCT  **(10mo)** | 2 | **Novel** |
| **41** | c.1276_1286del  p.(Asn426fs) | 12 | Other | Frameshift | Enteropathy  **(Birth)** | Enteropathy, skin disorder, thrombocytopenia  **(5w)** | Enteropathy, skin disorder, thrombocytopenia  **(5w)** | 3 | **Novel** |
| **42** | c.766A>G  p.(Met256Val) | 8 | Other | Missense | Diabetes  (**7w**) | Diabetes, rheumatic arthritis, eczema, eosinophilia, motor delays  (**4y**) | Diabetes, rheumatic arthritis, eczema, eosinophilia, motor delays, food allergies (**4y**) | 4 | [21] |
| **43** | c.1040G>A  p.(Arg347His) | 10 | Fork-head | Missense | Diabetes  (**2mo**) | Diabetes, polydactyl, delayed dentition (**15mo**) | Diabetes, polydactyl, delayed dentition (**2y**) | 1 | [1-4] |
| **44** | c. 210+2T>C  (p.?) | 2 | Splice site | Splice site | Diabetes  **(3w)** | Diabetes, chronic, severe malnutrition **(12y)** | Diabetes, chronic, severe malnutrition **(12y)** | 2 | **Novel** |
| **45** | c.751_753del  p.(Glu251del) | 8 | Leucine zipper | In-Frame  Deletion | Diabetes  (**10d)** | Diabetes, enteropathy **(6y)** | Diabetes, enteropathy **(6y)** | 2 | [8, 19] |
| **46** | c.1108A>G  p.(Met370Val) | 11 | Fork-head | Missense | Enteropathy **(unknown)** | Enteropathy, suspected immunodeficiency  **(8mo)** | Enteropathy, suspected immunodeficiency, atopic dermatitis **(9mo)** | 2 | RCV000414229.1  Conference abstract PMID: 28188499 |
| **47** | c.1222G>A  p.(Val408Met) | 12 | Fork-head | Missense | Diabetes  **(2d)** | Diabetes, nephrotic syndrome, TIA, peripheral hypothyroidism **(14y)** | Diabetes, nephrotic syndrome, transient ischemic attac, peripheral hypothyroidism **(14y)** | 2 | [8, 11] |
| **48** | c.1271G>A  p.(Cys424Tyr) | 11_12 | Fork-head | Missense | Diabetes **(unknown)** | Diabetes  **(15w)** | Diabetes  **(15w)** | 1 | [8] |
| **49** | c.1112T>C  p.(Phe371Ser) | 11 | Fork-head | Missense | Diabetes  (**3w**) | Diabetes  (**3w**) | Diabetes, eczema, enteropathy, failure to thrive  (**34w**) | 3 | **Novel** |
| **50** | c.1033C>A  p.(Leu345Ile) | 10 | Fork-head | Missense | Unknown | Membranous nephropathy, dairy intolerance, nodular hyperplasia, recurrent infections, failure to thrive, hypertension, cerebellar ataxia of uncertain origin  (**4y**) | Membranous nephropathy, dairy intolerance, nodular hyperplasia, recurrent infections, failure to thrive, hypertension, cerebellar ataxia of uncertain origin  (**4y**) | 2 | **Novel** |
| **51** | c.1015C>G  p.(Pro339Ala) | 10 | Fork-head | Missense | Diabetes  (**1w**) | Diabetes  (**6w**) | Diabetes, hypothyroidism  (**7w**) | 1 | [1, 11] |
| **52** | c.1190G>A  p.(Arg397Gln) | 12 | Fork-head | Missense | Diabetes  (**unknown**) | Diabetes  (**40w**) | Diabetes  (**40w**) | 1 | [17, 18] |
| **53** | c.1150G>A  p.(Ala384Thr) | 12 | Fork-head | Missense | Diabetes  (**1w**) | Diabetes  (**6w**) | Diabetes  (**6w**) | 1 | [15, 16] |
| **54** | c.305delT  p.(Phe102fs) | 3 | N-terminal | Frameshift | Diabetes  (**6mo**) | Diabetes  (**12y**) | Diabetes  (**12y**) | 1 | [22] |
| **55** | c.227delT  p.(Leu76fs) | 3 | N-terminal | Frameshift | Diabetes  (**1d**) | Diabetes, enteropathy, persistent respiratory infections  (**14w**) | Diabetes, neutropenia, thrombocytopenia, anemia, hyper IgE, enteropathy, hypothyroidism  (**Deceased aged 3.5y**) | 4 | [11] |
| **56** | c.1010G>A  p.(Arg337Gln) | 10 | Fork-head | Missense | Diabetes  (**30d**) | Diabetes  (**44d**) | Diabetes, enteropathy, hyper IgE, inflammatory tumor in ileon terminal  (**Deceased 1y 2mo)** | 3 | [8, 11-13] |
| **57** | p.(Arg347Cys) | 10 | Fork-head | Missense | Diabetes  (**11mo**) | Diabetes  (**unknown**) | Diabetes  (**unknown**) | 1 | **Novel** |
| **58** | c.1195G>A  p.(Glu399Lys) | 12 | Fork-head | Missense | Diabetes  (**11mo**) | Diabetes, IgA deficiency, muscle weakness, alopecia  (**17y**) | Diabetes, IgA deficiency, muscle weakness, alopecia, hypothyroidism  (**18y**) | 3 | **Novel** |
| **59** | c.1044+4A>G  p.(?) | 10 | Splice site | Splice site | Diabetes  (**5d**) | Diabetes  (**1mo**) | Diabetes, enteropathy, atopic dermatitis  (**Deceased aged 8mo**) | 3 | [23] |
| **60** | c.1271G>A  p.(Cys424Tyr) | 12 | Fork-head | Missense | Diabetes  (**7d**) | Diabetes, enteropathy, anaemia  (**Deceased aged 2mo)** | Diabetes, enteropathy, anaemia  (**Deceased aged 2mo)** | 3 | [8] |
| **61** | c.1234_1260del  p.(Glu412_Arg420del) | 12 | Fork-head | In-frame deletion | Diabetes  (**2mo**) | Diabetes, eczema, enteropathy, recurrent infections, anaemia, thrombocytopenia  (**3mo**) | Diabetes, eczema, enteropathy, recurrent infections, anaemia, thrombocytopenia  (**3mo**) | 5 | [24] |
| **62** | c.751_753del  p.(Glu251del) | 8 | Leucine zipper | In-Frame  Deletion | Diabetes  (**9mo**) | Diabetes, developmental delay, GI symptoms, autoimmune haemolytic anaemia, glomerulonephritis, AI hepatitis, chronic candidiasis, alopecia  (**3y 10mo**) | Diabetes, developmental delay, GI symptoms, autoimmune haemolytic anaemia, glomerulonephritis, AI hepatitis, chronic candidiasis, alopecia  (**3y 10mo**) | 5 | [8, 19] |
| **63** | c.967G>A  p.(Glu323Lys) | 9 | Other | Missense | Enteropathy  (**34d**) | Diabetes, enteropathy  (**38d**) | Diabetes, residual gastritis after HSCT  (**6y**) | 2 | [8] |
| **64** | c.1040G>A  p.(Arg347His) | 10 | Fork-head | Missense | Diabetes  (**8w**) | Diabetes  (**9w**) | Diabetes, elevated IgE, poor weight gain  (**2.5y**) | 2 | [1-4] |
| **65** | c.1157G>A  p.(Arg386His) | 12 | Fork-head | Missense | Enteropathy (**1w**) | Enteropathy, sepsis, hyper IgE, mild hepatitis, nephritis, eczema  (**4mo**) | Enteropathy, sepsis, hyper IgE, mild hepatitis, nephritis, eczema, lymphopaenia, thrombocytopaenia  (**10mo**)  HSCT 10mo – healthy aged **4y** | 5 | [8] |

***ESM Table 2:***

| **Presenting Feature** | **Additional Features Developed** | **Median age at latest follow-up (w)**  **(IQR)** |
| --- | --- | --- |
| **Diabetes**  (n=52) | Enteropathy  (n=22) | 56  (32-625.8) |
|  | Other (see ESM table 1)  (n=12) | 151.5  (21-847) |
|  | None  (n=14) | 25  (8.5-87) |
|  | Unknown  (n=4) | 15  (6-40) |
| **Enteropathy**  (n=11) | Diabetes  (n=5) | 162.9  (13-547.5) |
|  | Other (see ESM table 1)  (n=6) | 21.7  (9-43.5) |
| **Unknown**  (n=2) | Unknown  (n=2) | No data |

***ESM Table 3:*** Characteristics of patients with monogenic Tregopathies.

T1D-GRS = type 1 diabetes genetic risk score, presented as centile of 1800 type 1 diabetes controls from the Wellcome Trust Case Control Consortium. Y – years. W – weeks. D – days. LFT – liver function test.

| **ID** | **Age Collected** | **Age at**  **Onset** | **Additional Info** | **First feature** | **Genetic cause** | **T1D-GRS Percentile** | **Previously reported?** |
| --- | --- | --- | --- | --- | --- | --- | --- |
| **42** | 2y | 2d | Diabetes, low albumin, hypothyroidism | Diabetes | ***IL2RA***  c.418T>C/c.418T>C  p.(Tyr140His)/p.(Tyr140His) | 1.1 | No |
| **43** | 2w | 2w | Diabetes, developmental delay, hypothyroidism, jaundice | Diabetes | ***IL2RA***  c.710_711del/N  p.(Tyr41Cys)/N | 30 | No |
| **44** | 4y | 15mo | Diarrhea, hematological disorders - thrombopenia, adenopathy, splenomegaly | Diabetes | ***LRBA***  c.7042C>T/N  p.(Arg2348*)/N | 21 | Yes [25] |
| **45** | 10w | 7w | Diabetes, cleft lip | Diabetes | ***LRBA***  c.7976C.A/c.3156del  p.(Ser2659*)/p.(Asp1053fs) | 19.9 | Yes [25] |
| **46** | 42w | 37w | Diabetes, anaemia, malabsorption | Diabetes | ***LRBA***  c.3988dup/N  p.(Ile1330fs)/N | 21.7 | No |
| **47** | 26w | 23w | Abnormal LFTs, acute bowl infection and diarrhea | Diabetes | ***LRBA***  c.2836_2839del/N  p.(Glu946*)?N | 10.2 | No |
| **48** | 24y | 40w | Unretractable diarrhea, growth impairment, delayed puberty, severe vitamin D deficiency, iron deficiency, hypocalcaemia, hypophosphateamia | Diabetes | ***LRBA***  c.(4730+1_4730-1)_(5171+1_5171-1)del  c.4730-?_5171+?del/ c.4730-?_5171+?del | 58.7 | Yes [25] |
| **49** | 1y | 35w | Diabetes | Diabetes | ***LRBA***  c.129dup/N  p.(Ileu44fs)/N | 4.1 | No |
| **50** | 9y | 2y 2mo | Exocrine and endocrine pancreatic dysfunction, prolonged diarrhea, malnutrition enteropathy, coeliac disease | Diabetes | ***LRBA***  c.6267_6271delinsTTTT/N  p.(Ser2090fs)/N | 30 | No |
| **51** | 47w | 4mo | Diabetes, Evan syndrome, aggamaglobulinemia, lymphocytic interstitial pneumonia, generalised lymphadenopathy & hepatosplenomegaly - reactive lymphoproliferative disorder, recurrent chest infection - aspergillus pneumonia | Diabetes | ***LRBA***  c.3811C>T/c.3811C>T  p.(Arg1271*/p.(Arg1271*) | 3.7 | Yes [25] |
| **52** | 42w | 0w | Diabetes, developmental delay | Diabetes | ***STAT3***  c.1973A>T/N  p.(Lys658Met)/N | 54.4 | Yes [26] |
| **53** | 32w | 0 | Diabetes, anaemia | Diabetes | ***STAT3***  /N  p.(Gly421Arg)/N | N/A | No |
| **54** | 27w | 24w | Diabetes | Diabetes | ***LRBA***  /N  p.(Asp1132fs)/N | N/A | No |

***ESM Table 4: Characteristics of p.(Pro75Leu) pedigree (proband highlighted in green)***

| **ID** | **NM_014009.4 (FOXP3) variant** | **Exon/**  **Intron** | **Protein Effect** | **First feature** | **Clinical features at sampling** | **TSDR/CD4 (%)** |
| --- | --- | --- | --- | --- | --- | --- |
| **III.1** | c.224C>T  p.(Pro75Leu) | 3 | Missense | Diabetes | Diabetes | **23.5** |
| **III.2** | c.224C>T  p.(Pro75Leu) | 3 | Missense | Diabetes | Diabetes, failure to thrive, lymphadenopathy, hepatomegaly | 28.1 |
| **III.3** | N/N | N/A | N/A | Unaffected | Unaffected | 9.5 |
| **III.5** | c.224C>T  p.(Pro75Leu) | 3 | Missense | Diabetes | Diabetes, otitis media, bacterial meningitis, respiratory symptoms | 15.2 |
| **II.1** | c.224C>T  p.(Pro75Leu) | 3 | Missense | Diabetes | Diabetes, rheumatoid arthritis | 30.3 |
| **III.1,** | N/N | N/A | N/A | Unaffected | Unaffected | 10.1 |
| **III.5** | N/N | N/A | N/A | Unaffected | Unaffected | 10.2 |

***ESM Table 5: Classification of symptoms***

| **System** | **Conditions** |
| --- | --- |
| Endocrine | Diabetes  Hypothyroidism |
| Gut | Coeliac disease  Ulcerative colitis  Enteropathy |
| Epithelial | Eczema  Alopecia |
| Blood | Thrombocytopenia  Haemolytic anaemia  Hypochromic microcytic anaemia  Anaemia not specified |
| Immunological | Eosinophilia  Pneumonia  Recurrent infections  Hyper IgE |
| Other | Glomerulonephritis  Hepatosplenomegaly  Abnormal LFTs (Liver)  Rheumatoid arthritis |

# FIGURES

***
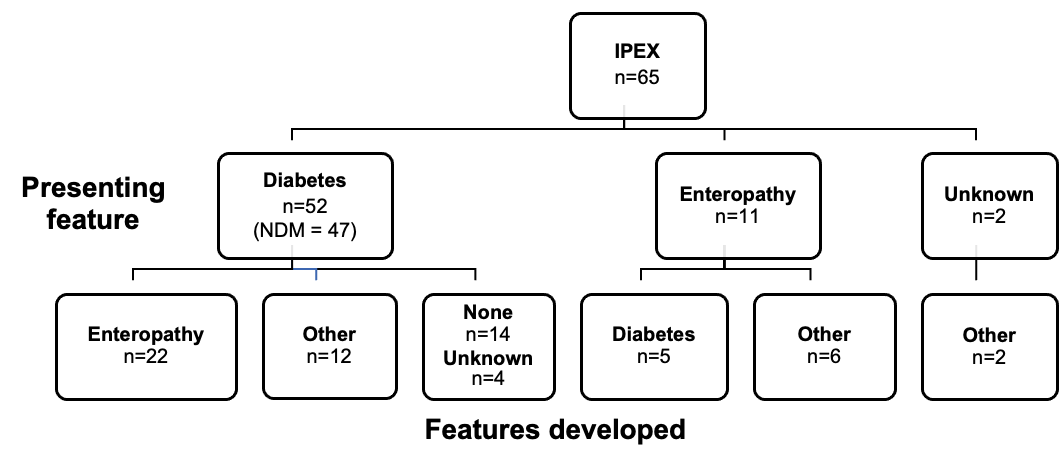
***

***ESM Fig. 1:*** IPEX cohort description

******

**ESM Fig. 2: Epigenetic quantification of cells with demethylation at the CD4 locus and the FOXP3 Treg Specific Demethylated Region in whole blood derived DNA from IPEX cases and controls.**

1. Cells with demethylation at the CD4 locus (%) in controls (n=29) and IPEX patients (n=41). The median values and IQR were 20% [14-32%] and 15.4% [11.9-20.3%], respectively, p=0.03. Horizontal lines represent the median and interquartile range.
2. Cells with demethylation at the TSDR locus (%) in controls (n=29) and IPEX patients (n=41). The median values and IQR were 2% [1-3%] and 2.4% [1.8-3.3%], respectively, p=0.05. Horizontal lines represent the median and interquartile range.

**ESM figure 3**: Age at latest follow-up (w) in patients with a TSDR/CD4 value of <13.4% (n=15, median 26.1w, IQR 13-886w) and ≥13.4% (n=18, median 43.5w, IQR 19.8-156.3, p=0.6). Horizontal lines represent the median and interquartile range.

# REFERENCES

1. Gambineri, E., et al., *Clinical and molecular profile of a new series of patients with immune dysregulation, polyendocrinopathy, enteropathy, X-linked syndrome: inconsistent correlation between forkhead box protein 3 expression and disease severity.* J Allergy Clin Immunol, 2008. **122**(6): p. 1105-1112.e1.

2. McMurchy, A.N., et al., *Point mutants of forkhead box P3 that cause immune dysregulation, polyendocrinopathy, enteropathy, X-linked have diverse abilities to reprogram T cells into regulatory T cells.* Journal of Allergy and Clinical Immunology, 2010. **126**(6): p. 1242-1251.

3. Seidel, M.G., K. Boztug, and O.A. Haas, *Immune Dysregulation Syndromes (IPEX, CD27 Deficiency, and Others): Always Doomed from the Start?* Journal of Clinical Immunology, 2016. **36**(1): p. 6-7.

4. Zama, D., et al., *Late-onset of immunodysregulation, polyendocrinopathy, enteropathy, x-linked syndrome (IPEX) with intractable diarrhea.* Italian Journal of Pediatrics, 2014. **40**: p. 7.

5. Luo, Y.Y., et al., *A case of Metaplastic atrophic gastritis in immune Dysregulation, Polyendocrinopathy, Enteropathy, X-linked (IPEX) syndrome.* Bmc Pediatrics, 2018. **18**: p. 5.

6. Hashimura, Y., et al., *Minimal change nephrotic syndrome associated with immune dysregulation, polyendocrinopathy, enteropathy, X-linked syndrome.* Pediatric Nephrology, 2009. **24**(6): p. 1181-1186.

7. Wildin, R.S., S. Smyk-Pearson, and A.H. Filipovich, *Clinical and molecular features of the immunodysregulation, polyendocrinopathy, enteropathy, X linked (IPEX) syndrome.* Journal of Medical Genetics, 2002. **39**(8): p. 537-545.

8. Gambineri, E., et al., *Clinical, Immunological, and Molecular Heterogeneity of 173 Patients With the Phenotype of Immune Dysregulation, Polyendocrinopathy, Enteropathy, X-Linked (IPEX) Syndrome.* Frontiers in Immunology, 2018. **9**: p. 18.

9. Li, B., et al., *FOXP3 is a homo-oligomer and a component of a supramolecular regulatory complex disabled in the human XLAAD/IPEX autoimmune disease.* International Immunology, 2007. **19**(7): p. 825-835.

10. Mailer, R.K., *IPEX as a Consequence of Alternatively Spliced FOXP3.* Frontiers in Pediatrics, 2020. **8**: p. 8.

11. Rubio-Cabezas, O., et al., *Clinical Heterogeneity in Patients With FOXP3 Mutations Presenting With Permanent Neonatal Diabetes.* Diabetes Care, 2009. **32**(1): p. 111-116.

12. Sheikine, Y., et al., *Renal involvement in the immunodysregulation, polyendocrinopathy, enteropathy, X-linked (IPEX) disorder.* Pediatric Nephrology, 2015. **30**(7): p. 1197-1202.

13. Savova, R., et al., *Clinical Case of Immune Dysregulation, Polyendocrinopaty, Enteropathy, X-Linked (IPEX) Syndrome with Severe Immune Deficiency and Late Onset of Endocrinopathy and Enteropathy.* Case Rep Med, 2014. **2014**: p. 564926.

14. Seghezzo, S., J.J. Bleesing, and Z.Y. Kucuk, *Persistent Enteropathy in a Toddler with a Novel FOXP3 Mutation and Normal FOXP3 Protein Expression.* Journal of Pediatrics, 2017. **186**: p. 183-185.

15. Wildin, R.S., et al., *X-linked neonatal diabetes mellitus, enteropathy and endocrinopathy syndrome is the human equivalent of mouse scurfy.* Nature Genetics, 2001. **27**(1): p. 18-20.

16. Bennett, C.L., et al., *The immune dysregulation, polyendocrinopathy, enteropathy, X-linked syndrome (IPEX) is caused by mutations of FOXP3.* Nature Genetics, 2001. **27**(1): p. 20-21.

17. Tsuda, M., et al., *The spectrum of autoantibodies in IPEX syndrome is broad and includes anti-mitochondrial autoantibodies.* Journal of Autoimmunity, 2010. **35**(3): p. 265-268.

18. Martin-Santiago, A., et al., *Diagnostic Value of the Skin Lesions in Immune Dysregulation, Polyendocrinopathy, Enteropathy, X-Linked Syndrome.* Pediatric Dermatology, 2013. **30**(6): p. E221-E222.

19. Consonni, F., S.C. Mannurita, and E. Gambineri, *Atypical Presentations of IPEX: Expect the Unexpected.* Frontiers in Pediatrics, 2021. **9**.

20. Bich, N.C.T., et al., *IPEX Syndrome Caused by A Novel Mutation in Foxp3 Gene: A Case Report.* Hormone Research in Paediatrics, 2016. **86**: p. 353-354.

21. Fang, Y., et al., *Atypical late-onset severe gastritis in immune dysregulation, polyendocrinopathy, enteropathy, and X-linked (IPEX) syndrome: 2 case reports.* Medicine (Baltimore), 2021. **100**(3): p. e24318.

22. Frith, K., et al., *The FOXP3Δ2 isoform supports Treg cell development and protects against severe IPEX syndrome.* J Allergy Clin Immunol, 2019. **144**(1): p. 317-320.e8.

23. Chatila, T.A., et al., *JM2, encoding a fork head-related protein, is mutated in X-linked autoimmunity-allergic disregulation syndrome.* The Journal of clinical investigation, 2000. **106**(12): p. R75-R81.

24. Ellard, S., et al., *Improved genetic testing for monogenic diabetes using targeted next-generation sequencing.* Diabetologia, 2013. **56**(9): p. 1958-1963.

25. Johnson, M.B., et al. *Recessively inherited LRBA mutations cause autoimmunity presenting as neonatal diabetes.* Diabetes, 2017. **66**(8): p 2316-2322

26. Flanagan S.E., et al. *Activating germline mutations in STAT3 cause early-onset multi-organ autoimmune disease*. Nat. Genet. 2014. **46**(8): 812-814
